# Polypharmacy and Medication-Related Problems in Hemodialysis Patients: A Call for Deprescribing

**DOI:** 10.3390/pharmacy6030076

**Published:** 2018-07-25

**Authors:** Majed Alshamrani, Abdullah Almalki, Mohamed Qureshi, Oyindamola Yusuf, Sherine Ismail

**Affiliations:** 1 King Abdullah International Medical Research Center, King Saud Bin Abdulaziz University for Health Sciences, Pharmaceutical Care Department, King Khalid Hospital, Ministry of National Guard Health Affairs, Jeddah 21423, Saudi Arabia; ShamraniMA01@ngha.med.sa; 2 King Abdullah International Medical Research Center, King Saud Bin Abdulaziz University for Health Sciences, Department of Medicine, Nephrology Section, King Khalid Hospital, Ministry of National Guard Health Affairs, Jeddah 21423, Saudi Arabia; MalkiA02@ngha.med.sa (A.A.); QureshiMA@ngha.med.sa (M.Q.); 3 Department of Epidemiology and Medical Statistics, College of Medicine, University of Ibadan, Ibadan, Nigeria; bidemiyusuf1@gmail.com

**Keywords:** drug-related problems, medication-related problems, pharmacist, polypharmacy, hemodialysis

## Abstract

Polypharmacy is a common problem among hemodialysis patients. It is associated with increased hospital admissions, morbidity, mortality, Medication-Related Problems (MRPs), and expenditures. There is a paucity of data on the prevalence of polypharmacy in our setting. This study aims to determine the prevalence of polypharmacy and MRPs and to assess its predictors. We conducted a cross-sectional study in the outpatient hemodialysis unit. A pharmacy resident assessed electronic prescribing records to identify MRPs and discussed therapeutic interventions to enhance effective therapeutic regimens over a three months period. Eighty-three patients were included. The median age was 63 (Interquartile range; IQR = 22), 50% were males, and the mean number of co-morbidities was 3.14 ± 1.64. The prevalence of polypharmacy was 97.6% with a 95% CI (91.6%–99.7%). Medication use without indication, was the highest identified MRPs at 36% (102/280), followed by subtherapeutic dosing at 23% (65/280), and overdosing at 15% (41/280). The number of comorbidities, the presence of ischemic heart disease, and respiratory diseases were the main predictors of the increased number of medications. Polypharmacy is highly prevalent among the Saudi hemodialysis population. A review of the medications prescribed by the pharmacist facilitated the identification of MRPs and provided opportunities for deprescribing to optimize medication use and to reduce polypharmacy in hemodialysis patients.

## 1. Introduction

End-Stage Renal Disease (ESRD) is a worldwide public health problem [1]. In 2014, the incidence of newly diagnosed dialysis patients was 4177 of Saudi Arabia’s total population of 30,770,475; the prevalence was 136 PMP (Per Million Population) [2]. According to the Saudi Centre for Organ Transplantation (SCOT) registry data, the total number of patients undergoing hemodialysis (HD) was 16,315 by the end of 2016 [3].

ESRD patients have multiple complications related to kidney failure, such as; Fluid overload hypertension; anemia; secondary hyperparathyroidism; and, uremic pruritus, in addition to other chronic comorbidities, which require the use of several medications and carry an increased associated risk of medication errors [4]. Additionally, Saudi Arabia’s elderly population is growing alongside a projected increase in the prevalence and incidence of dialysis, which further contributes to the complexity of prescribing medications to those patients [5]. Furthermore, dialysis patients have a mean of 4.7 medications with a range of 2–9 healthcare providers—excluding nephrologists and the utilization of over the counter medications (OTC)—which explains the high rate of polypharmacy in this population [6].

Several reports have defined polypharmacy, in the literature, as the use of ≥4–5 medications, regardless of a clinical indication, which may lead to several Medication-Related Problems (MRPs). Hence, patient safety is compromised [7,8,9]. Several categories of MRPs were described in chronic kidney disease patients, including; (1) untreated indications; (2) improper drug selection; (3) improper drug dosing; (4) adverse drug reactions; (5) drug–drug interactions; (6) adherence; and, (7) drug use without indication [10]. MRPs may increase hospital admissions, morbidity, mortality, and pose a financial burden to the healthcare system [4]. Therefore, some strategies have been reported, in the literature, to minimize polypharmacy and to optimize the adherence of patients to their medications and dietary regimens such as multidisciplinary team rounds, including pharmacists [11,12]. 

Pharmacists are trained to identify MRPs through a structured review of a patient’s medication list and to communicate with physicians to find therapeutic alternatives, or, to deprescribe unnecessary medications in order to reduce polypharmacy [13,14,15,16,17,18]. Several studies established the crucial role of pharmacists in identifying MRPs associated with polypharmacy and suggested appropriate interventions to optimize patient outcomes [14,15,19].

To the best of our knowledge, there is a paucity of data on the prevalence of polypharmacy among Saudi hemodialysis patients, and its impact on MRPs. Therefore, this study aims to determine the prevalence of polypharmacy and the Medication-Related Problems in hemodialysis patients at King Abdulaziz Medical City, Jeddah.

## 2. Materials and Methods 

### 2.1. Design and Setting 

A cross-sectional study was conducted in the outpatient hemodialysis unit of King Abdulaziz Medical City, Jeddah, Saudi Arabia.

### 2.2. Study Population

Patient records were eligible for inclusion if patients were older than 18 years of age and were undergoing hemodialysis three times per week, at the same dialysis unit, during the study period. Hemodialysis patients who were admitted to the hospital as inpatients at the time of data collection were excluded. 

### 2.3. Methods 

Data collection was done by: Reviewing patient charts; electronic medical records, for medications; and, through discussion with the treating physicians by the pharmacy resident from December 2010 to February 2011.

The pharmacy resident reviewed electronic medical records to assess the number of prescribed medications and to identify baseline demographics, comorbidities and clinical laboratory data. Medications were documented in our electronic records using the generic name(s) for the active ingredient(s) and medications containing combinations were considered as single drugs. In addition, each medication regimen for eligible patients were analyzed to identify MRPs and were evaluated to classify the severity of drug–drug interactions based on the classification of the Micromedex^®^ drug information databases. The analysis was based on the appropriateness of: Dosing; duplication of therapy; adverse drug events; contraindications for use; and, whether laboratory parameters were required for the monitoring of drugs and the appropriateness for use based on the clinical history documented by physicians in the medical charts and laboratory results reported in the electronic healthcare system. The severity of drug–drug interactions were classified as minor, moderate, major, and contraindicated. They are defined as follows:

(1) Minor: Limited clinical effects and does not require therapy modification
(2) Moderate: The interaction may result in the exacerbation of the patient’s condition and/or requires therapy modification
(3) Major: The interaction may be life-threatening and/or requires medical intervention to minimize or prevent serious adverse events
(4) Contraindicated: The drugs are contraindicated for current use

We focused on the potentially important drug–drug interactions in our study, which required interventions such as moderate, major and contraindicated. The pharmacy resident discussed with the attending physician the suggested therapeutic interventions, such as: Discontinuing medications; altering medication dose; the route of administration; and, switching to new alternative medications. These therapeutic interventions, if accepted by the attending physicians, were implemented through prescribing medication orders to optimize patient medication regimens. Subsequently, the number of accepted interventions was recorded. The pharmacy resident and the three attending physicians were not changed throughout the study period.

### 2.4. Study Outcomes

The primary outcome was the prevalence of polypharmacy among outpatient hemodialysis patients. We defined Polypharmacy as receiving more than five prescribed medications per day at the time of data collection. 

Secondary outcomes included different types of MRPs, as was reported in previous studies. These include: Medication use without indication; improper drug dosing, either subtherapeutic dosing or overdosing; indication without treatment; duplicate medications; contraindications; laboratory tests required for monitoring; alternative medications recommended; and, drug–drug interaction [17,20,21]. The drug–drug interactions requiring further action by the prescriber were included under the respective category of MRPs. Additional secondary outcomes include the number and proportion of suggested interventions, which were accepted by the treating physicians and the determinants of the mean number of medications.

### 2.5. Sample Size 

We included all accessible patients in our dialysis unit who fulfilled the eligibility criteria.

### 2.6. Statistical Analysis

The prevalence of polypharmacy is presented as a percentage, with a 95% confidence interval using an exact binomial test. We tabulated descriptive statistics by a count or percentage for categorical variables and either the mean ± standard deviation (SD), median, or interquartile range, (IQR) when appropriate, was used for continuous data. We used multiple linear regression to assess the determinants of the mean number of medications, as the dependent variable and number of comorbidities, age, gender, underlying cause of end stage renal disease (ESRD), and most common comorbidities were independent predictors. The model assumptions—independence, normality, homoscedasticity, and linearity—were assessed; Cook’s distance was calculated to identify influential observations on model fitness.

Statistical tests were conducted using a 5% level of significance. All analyses were carried out using STATA 14 (StataCorp LP, College Station, TX, USA).

### 2.7. Ethics 

The study received approval from the institutional review board of King Abdullah International Medical Research Center (KAMIRC) on 5 December, 2010.

## 3. Results

### 3.1. Population 

A total of 90 patients were screened for eligibility, of which, 83 met inclusion criteria. Seven patients were excluded, of which, four had hemodialysis twice weekly, and three were hospitalized during the study period. 

### 3.2. Baseline Characteristics 

The median age of our patients was 63, with an interquartile range of (49–71), and 50% of the study participants were male. The majority of our patients had two or three comorbidities, identified in 45 out of 83 patients (54.2%), and more than three comorbidities, detected in 28 out of 83 patients (33.7%). The mean number of medications in our cohort was 14 ± 4.6. Further details of baseline characteristics are presented in Table 1.

### 3.3. Study Outcomes 

#### 3.3.1. Primary Endpoint

The prevalence of Polypharmacy was 97.6% and 95% CI (91.6% to 99.7%). Only 2 out of 83 patients, had five medications.

#### 3.3.2. Secondary Endpoints

A total of 184 drug–drug interactions were identified, they had the following classification for the severity of interactions: Moderate 58% (106); major 41% (76); and, contraindicated 1% (2). The top medication classes, which were involved in drug–drug interactions, were: Antidepressants 10% (19); antiplatelet agents 8% (15); proton pump inhibitors 7% (13); and, statins 2% (4). 

The pharmacy resident suggested a total of 280 interventions for all 280 identified Medication-Related Problems. The median number of suggested interventions per patient was three (IQR = 2), and the median number of accepted interventions per patient was one (IQR = 1). The total number of accepted interventions was 130 out of 280 suggested interventions (46.43%). 

**Figure 1:**
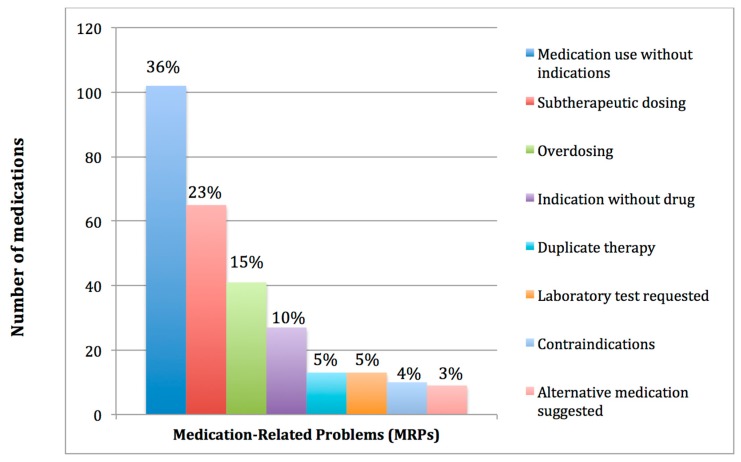
Frequency and types of Medication-Related Problems.

The most common Medication-Related Problem identified, among all suggested interventions, was medication use without indication 36% (102/280). This included: Proton pump inhibitors 15% (15/102); laxatives 14% (14/102); antiplatelet agents 11% (11/102); and, statins 8% (8/102). The second class of MRPs was subtherapeutic dosing, 23% (65/280), followed by overdosing, 15% (41/280). Figure 1 presents the frequency of different types of Medication-Related Problems.

Deprescribing of medications was required in 41% (115) of the suggested interventions for the following categories of MRPs: Medication use without indication 89% (102/115); and, duplicate therapy 11% (13/115).

Female gender, the number of comorbidities, the presence of ischemic heart disease and respiratory disease were the most statistically significant predictors identified, in the model for the multiple linear regression, for the number of medications, with a *p*-value of 0.026, 0.005, 0.003 and 0.026, respectively.

Diagnostics for model assumptions were met and revealed independence, linearity, normality, and homoscedasticity. There was one observation, which had the largest Cook’s distance; it was for a patient who had six comorbidities. Subsequently, the fitness of the model slightly improved, after removing this observation, with an adjusted R^2^ from 52.3% to 56.5%.

Furthermore, the number of comorbidities, the presence of ischemic heart disease and respiratory disease were the most statistically significant predictors identified in the final model.

Table 2 presents the regression coefficients and the 95% confidence intervals (CI) of all predictors included in the model after the exclusion of the observation with the most substantial Cook’s distance.

The majority of medications identified for respiratory diseases were inhalers for the treatment of asthma or chronic obstructive pulmonary disease, while statins, antiplatelet agents, medications to block Renin-Angiotensin System and β-blockers were the most commonly utilized medications for the treatment of ischemic heart disease.

## 4. Discussion

Our study’s findings have demonstrated that polypharmacy is highly prevalent among hemodialysis patients, with a mean number of medications at 14 ± 4.6. These results are consistent with previous reports of the average number of medications for dialysis patients, with 5–14 medications [6,21,22]. The large number of medications in our population could be explained by the mean number of comorbidities, 3.14 ± 1.64, which, by definition, leads to polypharmacy [5,20]. In addition, prescribing medications without indications had the highest frequency of MRPs at 36%, which magnified polypharmacy in our cohort.

In our study, medication review by the pharmacy resident led to 280 interventions in 83 hemodialysis patients during three months, which is similar to earlier reports. For example, a pooled analysis of seven studies, which included a total of 395 patients requiring HD, for whom pharmacists undertook medication review over approximately three-months, demonstrated an average number of 11.8 ± 0.7 (medication/patient). This analysis resulted in a total of 1593 MRPs [21]. Additionally, Mirkov et al. has shown that pharmacist-led medication review facilitated the identification of 287 MRPs and led to 493 interventions in 64 chronic HD patients over a period of six-months [23]. Similar to our study, these patients were prescribed an average of 13 medications, which may partly explain the high rate of MRPs identified in 92% of all reviewed medications [23]. 

The most frequent MRPs identified in our study was medication use without indications, with a rate of 36%, which is consistent with Belaiche et al. study that reported the highest percentage (31.7%) for the same category of MRPs. This demonstrates the crucial need for regular medication review and deprescribing processes [24]. Additionally, incorrect dosing was reported to be the second highest MRPs with 19%, which is comparable to our study with 23% for subtherapeutic dosing, and 15% for overdosage. However, Belaiche et al. included 67 chronic kidney disease patients at stage three-to-four who were not on hemodialysis [24]. Our findings were slightly different from Manley et al., which included a pooled analysis of studies including 395 dialysis patients where the requirement for laboratory monitoring was the most frequent MRPs (23.5%), followed by indication without drug (16.9%), and drug use without indications as the third MRPs (14.9%) [21]. The discrepancy in the percentage of MRPs could be explained by the different prescribing pattern, the various healthcare settings and the medical insurance status of patients.

In our study, three variables were identified as predictors of the number of medications used among chronic hemodialysis patients. These were: The number of comorbidities; the presence of ischemic heart disease; and, respiratory disease. These findings are consistent with Payne et al., which assessed the prevalence of polypharmacy in Scottish records for primary health care; the number of comorbidities and cardiovascular disease was reported as the most common predictors of polypharmacy [25]. Furthermore, similar to the findings of our multiple linear regression model, excluding one observation with the most substantial Cook’s distance, female gender was not found to be an independent predictor for polypharmacy in dialysis patients [25]. Finally, cardiovascular diseases are known to be the most common cause of morbidity and mortality in dialysis patients, which often requires the use of several classes of medications including angiotensin-converting enzyme inhibitors, β-blockers, statins and antiplatelet agents. Hence, the higher rate of polypharmacy in this population is explained [26,27]. 

There are several limitations to our study. First, the number of medications for every patient was based on a review of electronic medication records, rather than interviewing patients, which might have lead to inaccurate information about the actual number of medications received by the patient—such as the use of over the counter medications and/or non-adherence. In addition, a patient interview might have revealed additional opportunities for effective therapeutic interventions to deprescribe unnecessary medications and to reduce polypharmacy. Second, the duration of the study was short and the lack of a follow-up to assess the long-term impact of the pharmacist’s intervention to polypharmacy or the reduction of MRPs. Third, we reported a low acceptance rate for the suggested interventions as the resident pharmacist conducted the review of medications at an early stage of the residency training program, which demonstrates the importance of clinical experience and effective communication by pharmacists to enhance the acceptance rate of interventions by physicians. Furthermore, many of the rejected interventions required referrals to other specialists to re-evaluate the specific patient’s needs, which led to the reluctance of our physicians to accept these interventions. 

Our study has several strengths, to our knowledge, this is the first study in our region to characterize polypharmacy in dialysis patients and we assessed the most common predictors for the increasing number of medications among patients requiring hemodialysis. This provides insights for healthcare providers to optimize the use of pharmaceutical care services for the care of this unique population. In addition, the findings of our study support the crucial role of pharmacists in reviewing medications for dialysis patients on a regular basis to reduce polypharmacy, which is consistent with previous data [22]. A recently published study demonstrated the positive impact of targeted deprescribing of medications, due to the uncertainty of evidence in regards to their efficacy or safety, to minimize polypharmacy in outpatients requiring hemodialysis [28].

Future studies should assess the impact of medication therapy management services, expand on the evaluation of the targeted deprescribing of medications on a regular basis and at the transition of care during hospitalizations, to minimize polypharmacy and improve clinical outcomes in patients requiring hemodialysis [29,30].

## 5. Conclusions 

Polypharmacy is highly prevalent among the Saudi hemodialysis population. A structured review of the medications prescribed by pharmacists is necessary to identify MRPs and provides opportunities for deprescribing in order to optimize medication use and reduce polypharmacy in hemodialysis patients.

## Figures and Tables

**Table 1:** Baseline characteristics.

Baseline Characteristics	N = 83 ^a^
Age, median (IQR) ^b^	63 (49–71)
Gender (Males)	42 (51%)
Body mass index (kg/m^2^) ^c^, median (IQR)	23.87 (28.3–21.5)
Underlying cause of end stage kidney disease	
Hypertension	17 (21%)
Diabetes	11 (13%)
Diabetes and hypertension	20 (24%)
Glomerular diseases	2 (2%)
Other	11 (13%)
Unknown	22 (27%)
Number of comorbid conditions per patient, mean ± SD ^d^	3.14 ± 1.64
Comorbidities ^e^	
Hypertension	77 (93%)
Diabetes Mellitus	45 (54%)
Ischemic heart disease	31 (37%)
Dyslipidemia	21 (25%)
Cerebrovascular accident	12 (15%)
Respiratory diseases	11 (13%)
Thyroid disorders	6 (7%)
Epilepsy	6 (7%)
Hepatitis C virus infection	5 (6%)
Heart failure	5 (6%)
Gastrointestinal diseases	5 (6%)
Atrial fibrillation	4 (5%)
Peripheral vascular disease	3 (4%)
Depression	3 (4%)
Hepatitis B virus infection	1 (1%)

^a^ Unless otherwise indicated: Numbers present n (%); ^b^ IQR, interquartile range; ^c^ (kg/m^2^), kilogram/meter^2^, and, SD, standard deviation. ^e^ The list of comorbidities is exhaustive for our cohort and was identified as documented in the medical records.

**Table 2:** Regression coefficient and 95% CI for multiple linear regression analysis for predictors of the number of medications in chronic hemodialysis patients ^a^.

Variable	Β ^b^	95% Confidence Interval	*p*-Value
Age	0.03	−0.02, 0.08	0.278
Gender			
Female	Reference	-	-
Male	−1.39	−2.90, −0.12	0.070
Cause of hemodialysis			
Unknown	Reference	-	-
Hypertension	0.06	−2.19, 2.06	0.952
Diabetes	2.00	−0.72, 4.71	0.147
Hypertension and Diabetes	1.65	−0.63, 3.93	0.152
Glomerular disease	−0.91	−5.78, 3.95	0.709
Other	0.06	−2.45, 2.33	0.961
Number of comorbid conditions	1.16	0.45, 1.87	0.002 ^c^
Hypertension	0.12	−3.06, 3.30	0.941
Diabetes mellitus	−0.44	−2.57, 1.69	0.680
Ischemic heart disease	3.03	1.27, 4.97	0.001 ^c^
Cerebrovascular stroke	−0.33	−2.68, 2.03	0.782
Dyslipidemia	0.00	−2.02, 2.03	0.996
Respiratory disease	3.50	1.18, 5.84	0.004 ^c^

^a^ This model excludes the observation with the largest’s Cook’s distance: ^b^ β: Estimated regression coefficient; ^c^ statistically significant.
